# Involvement of the Ventrolateral Periaqueductal Gray Matter-Central Medial Thalamic Nucleus-Basolateral Amygdala Pathway in Neuropathic Pain Regulation of Rats

**DOI:** 10.3389/fnana.2020.00032

**Published:** 2020-07-21

**Authors:** Yi Sun, Jian Wang, Shao-Hua Liang, Jun Ge, Ya-Cheng Lu, Jia-Ni Li, Yan-Bing Chen, Dao-Shu Luo, Hui Li, Yun-Qing Li

**Affiliations:** ^1^Department of Human Anatomy, School of Basic Medical Sciences, Fujian Medical University, Fuzhou, China; ^2^Department of Cardiovascular Surgery, the General Hospital of Western Theater Command, Chengdu, China; ^3^Department of Human Anatomy, Binzhou Medical University, Yantai, China; ^4^Department of Anatomy, Histology and Embryology and K.K. Leung Brain Research Centre, Air Force Medical University, Xi’an, China; ^5^Key Laboratory of Brain Science Research and Transformation in Tropical Environment of Hainan Province, Haikou, China; ^6^Department of Human Anatomy, College of Basic Medicine, Dali University, Dali, China

**Keywords:** central medial nucleus, periaqueductal gray matter, basolateral nucleus of the amygdala, pathway, neuropathic pain, rat

## Abstract

The central medial nucleus (CM), a prominent cell group of the intralaminar nuclei (ILN) of the thalamus, and the ventrolateral periaqueductal gray matter (vlPAG) are two major components of the medial pain system. Whether vlPAG and CM are input sources of nociceptive information to the basolateral amygdala (BLA) and whether they are involved in neuropathic pain regulation remain unclear. Clarifying the hierarchical organization of these subcortical nuclei (vlPAG, CM, and BLA) can enhance our understanding on the neural circuits for pain regulation. Behavioral test results showed that a CM lesion made by kainic acid (KA) injection could effectively alleviate mechanical hyperalgesia 4, 6, and 8 days after spared nerve injury (SNI) surgery, with the symptoms returning after 10 days. Morphological studies revealed that: (1) the CM received afferents from vlPAG and sent efferents to BLA, indicating that an indirect vlPAG–CM–BLA pathway exists; (2) such CM–BLA projections were primarily excitatory glutamatergic neurons as revealed by fluorescence *in situ* hybridization; (3) the fibers originated from the CM-formed close contacts with both excitatory and inhibitory neurons in the BLA; and (4) BLA-projecting CM neurons expressed Fos induced by SNI and formed close contacts with fibers from vlPAG, suggesting that the vlPAG–CM–BLA indirect pathway was activated in neuropathic pain conditions. Finally, the vlPAG–CM–BLA indirect pathway was further confirmed using anterograde and monosynaptic virus tracing investigation. In summary, our present results provide behavioral and morphological evidence that the indirect vlPAG–CM–BLA pathway might be a novel pain pathway involved in neuropathic pain regulation.

## Introduction

Neuropathic pain is caused by disorders of, or damage to, the nervous system. Its refractory nature and the lack of effective treatment strategies make it a major challenge (Ciaramella, 2019). Neuropathic pain can cause central sensitization of the nervous system; however, the subcortical modulation pathways of neuropathic pain have not been clearly elucidated.

The intralaminar nuclei (ILN) of the thalamus are thought to comprise an important portion of the ascending reticular activating system and are necessary for the maintenance of the state of consciousness (von Cramon, 1978). Recent studies have demonstrated that the central medial (CM) nucleus, an essential component of ILN, is related to trigemino-vascular headaches and visceral pain (Ter Horst et al., 2001; Lazovic et al., 2005; Zhang et al., 2009). In addition, most of the ILN neurons, including the CM, responded to both peripheral innocuous and noxious stimuli and mediated the nociceptive short-term plasticity through the CM–anterior cingulate cortex (ACC) pathway in rats. In humans, nociceptive neuron activities have been recorded within the CM *via* electrophysiological recordings (Konietzny et al., 2016). However, no direct behavioral or morphological evidence proved that the CM is involved in neuropathic pain processing, and the underlying mechanism requires further investigation.

The basolateral amygdala (BLA) was identified to be critical for the development of neuropathic pain and depressive-like behaviors (Seno et al., 2018; Huang et al., 2019). The BLA receives polymodal sensory and nociceptive information mainly from the brain stem and cortical systems (Neugebauer, 2015). In addition, dense CM projections to the amygdala selectively target the BLA (Turner and Herkenham, 1991). Moreover, several studies have focused on the CM–ACC pain pathway, but not the CM–BLA pathway (Shyu and Vogt, 2009). Therefore, whether CM is another important source of pain information for the BLA remains unknown.

The ventrolateral quadrant of the periaqueductal gray matter (vlPAG) responds specifically to somatic nociceptive stimuli (Sanders et al., 1980). Recent studies of the vlPAG have focused on its role in the descending pain pathway (Huang et al., 2019; Lau et al., 2020). However, vlPAGs ascendingly project to the intralaminar thalamic nuclei in rats, including the CM, parafascicular, paracentral, and central lateral nuclei (Krout and Loewy, 2000). Moreover, it has been proposed that vlPAG and ILN are two subcortical structures that mediate the motivational aspect of pain (Sewards and Sewards, 2002). Open literature to date has not reported that the vlPAG–CM pathway is an ascending pain pathway under neuropathic conditions. Therefore, whether CM receives pain information from vlPAG remains to be investigated.

To clarify the communications among vlPAG, CM, and BLA, we hypothesized that there was an indirect vlPAG–CM–BLA ascending pathway. This pathway might be involved in neuropathic pain modulation as an important source of nociceptive information for the BLA. To test this hypothesis, behavioral and morphological investigations were conducted in the present study.

## Materials and Methods

### Animals

Adult male Sprague–Dawley rats (weighing between 250 and 300 g) were obtained from the Ethics Committee of the Air Force Medical University (Xi’an, China). Rats were housed in a 12-h light/dark cycle environment and provided free access to food and water. The protocols were approved by the Air Force Medical University. The number of rats used was as little as possible, and the suffering was minimized according to International Association for the Study of Pain guidelines (Zimmermann, 1983).

### Brain Lesion and Groups

Rats for behavioral tests were randomly divided into five groups as follows: (1) normal control (CON) group: rats were not disturbed in their cages (*n* = 6); (2) kainic acid (KA) group: rats received KA ablation of the CM only (*n* = 6); (3) spared nerve injury (SNI) group: the rats underwent SNI only (*n* = 10); (4) SNI + Saline group: the rats received SNI surgery and an injection identical in volume to that of the KA injection of sterile 0.9% saline into the CM (*n* = 6); and (5) SNI + KA group: rats received SNI surgery and KA ablation of the CM (*n* = 7). The rats were allowed to survive for 30 days. All rats were anesthetized with 2% sodium pentobarbital (40 mg/kg, intraperitoneal) and fixed on a stereotaxic apparatus (68,025, RWD, Shenzhen, China). KA was slowly injected into the target brain areas using a 1-μl Hamilton syringe. KA is widely used as an excitatory neurotoxic drug in lesions of the nuclei in the brain (Aguilar-Arredondo et al., 2017). The CM was ablated with KA (1.0 μg/μl, 0.2 μl) with saline injected as control.

### Tracer Injection and Groups

All rats were anesthetized with 2% sodium pentobarbital (40 mg/kg, intraperitoneal) and fixed on a stereotaxic apparatus. Tract tracings were performed with different rats from those used in the behavioral experiment. Retrograde tracer Fluoro-Gold (FG, 4% [w/v], 80014, Biotium, Hayward, CA, USA) dissolved in deionized water was injected into the targeted brain areas. Plant lectin, *Phaseolus vulgaris* leucoagglutinin (PHA-L, 4% [w/v], L1110, Vector Labs, Burlingame, CA, USA), dissolved in 0.9% (w/v) saline buffer was iontophoretically deposited (positive pulses, 4 μA, 7 s “on”/7 s “off,” 30 min) into the nuclei, *via* a glass micropipette (tip diameter 50 μm) with a voltage stimulator. The procedures for tracer injection were identical to those of our previous studies (Wang et al., 2014). The head of each rat was fixed in a stereotaxic apparatus. One or two small holes were made in the right side of the skull with a dental drill.

To investigate the neuronal properties of the BLA of the CM–BLA pathway, 60 nl FG was injected into the medial prefrontal cortex (mPFC), and PHA-L was iontophoretically injected into the CM. Rats were allowed to survive for 7 days and followed double-immunofluorescent histochemical staining for PHA-L/PV, PHA-L/FG, and PHA-L/VGluT2, respectively (*n* = 8).

To verify the presence of the vlPAG–CM–BLA pathway and to observe Fos protein expression within the CM, 40 nl FG was injected into the BLA, and PHA-L was iontophoretically injected into the vlPAG (*n* = 16). Rats were divided into two groups (sham and SNI-Fos group). In eight of the rats (sham group), the three branches of the sciatic nerve were simply exposed, and the deep fascia and skin were then sutured closed. Fluorescence *in situ* hybridization (FISH) with FG immunohistochemical staining and triple-immunofluorescent histochemical staining for PHA-L/FG/FOS were performed. The remaining eight rats (SNI-Fos group) received tracer injections as above and were subjected to SNI surgery before the skin was sutured closed. Triple-immunofluorescent histochemical staining for PHA-L/FG/FOS was performed. FG and PHA-L injection rats were allowed to survive for 7 days.

A two-step tracing was used to further examine the vlPAG–CM–BLA pathway (*n* = 3). Red Retrobeads (R170, Lumafluor Inc., UT, USA) were previously diluted in PBS at a ratio of 1:500. A mixture (500 nl) of Cre-dependent anterograde trans-monosynaptic virus AAV2/1-hSyn-Cre-WPRE-PA (2.37E + 13V.G./ml, S0278, Tailtool Bioscience, Shanghai, China) and Red Retrobeads (2% [v/v]; ratio 1:1) was injected into vlPAG, while the CM was injected with 200 μl AAV2/9-hSyn-DIO-eGFP (1.61E + 13V.G./ml, S0270-9, Tailtool Bioscience, Shanghai, China). The Red Retrobeads were added to indicate whether the colorless trans-synaptic virus AAV2/1-hSyn-Cre-WPRE-PA was properly injected into the vlPAG. Virus-injected rats were allowed to survive for 30 days. The coordinates of the brain structures were based on the atlas of the rat brain by Paxinos and Watson (Paxinos et al., 1985).

Injection coordinates were shown as follows: mPFC (anteroposterior 3.7 mm, lateral to midline 0.8 mm, dorsoventral 3.6 mm); CM (anteroposterior −2.6 mm, lateral to midline 0 mm, dorsoventral 6.2 mm); BLA (anteroposterior −2.2 mm, lateral to midline 4.9 mm, dorsoventral 8.6 mm); and vlPAG (anteroposterior −7.56 mm, lateral to midline 0.7 mm, dorsoventral 6.2 mm). The incision was closed using 4–0 silk sutures. All injections were delivered at a speed of 10 nl/min to limit the infection range, and the glass micropipette was left in place for 15 min to minimize leakage.

### SNI Surgery

SNI surgery was conducted as previously reported (Pertin et al., 2012). Briefly, rats were anesthetized with 2% sodium pentobarbital (40 mg/kg, intraperitoneal). The skin along the back of the thigh was incised. The deep fascia and femoral biceps were separated, exposing the three branches of the sciatic nerve. The tibial and common peroneal nerves were tightly ligated with 4.0 silk, and the part of the nerve distal to the ligation was sectioned to remove 3 mm of the distal nerve. The sural nerve was left intact, and the deep fascia and skin were closed.

### Mechanical Paw Withdrawal Threshold (PWT) Analysis

Animals were acclimated to the environment for 3 days prior to experimentation. Rats were placed in a transparent plastic box (250 × 250 × 350 mm) on an elevated metal wire grid. After the rats acclimatized for a period of 30 min, the lateral plantar surface of the paw was stimulated with a series of Von Frey filaments (Aesthesio, Danmic Global, CA, USA) of increasing force. The mechanical paw withdrawal threshold (PWT) was taken as the lowest force that evoked a brisk withdrawal response to three of five repetitive stimuli. The baseline of PWT measurement was performed 1 day before the CM lesion, and the behavioral test was also performed on days 4, 6, 8, 10, 12, 14, 20, and 28 after SNI operation.

### Perfusion and Immunohistochemical Staining

After deep anesthesia with 2% sodium pentobarbital (100 mg/kg, intraperitoneal), the rats in the behavioral groups were perfused transcardially with 150 ml 0.01 M PBS, pH 7.4, followed by 500 ml 4% (w/v) paraformaldehyde in sodium phosphate buffer (0.1 M PB, pH 7.4) for 90 min. The brains were removed and postfixed for 4 h, all in the same fixative, and then immersed in 30% (w/v) sucrose in 0.1 M PB for 48 h at 4°C. The brains were sectioned into 40-μm slices using a cryostat (CM1950, Leica, Heidelberg, Germany) at −20°C. For the Nissl staining, slices from each group of rats were defatted overnight by 70% (v/v) ethanol at 37°C and then immersed in crystal violet solution for 1–3 min until reaching the required depth of staining. The slices were then washed with distilled water, dehydrated in 70, 80, 90, 95, and 100% (v/v) ethanol for several seconds each, cleared in xylene for 2 h, mounted with neutral gum, and coverslipped. The sections were observed under an optical microscope (AHBT3, Olympus, Tokyo, Japan).

The avidin–biotin–peroxidase complex (ABC) method was used to stain sections of the injection site, which included the BLA, the CM, and the vlPAG. The sections were blocked with 10% donkey serum in 0.01 M phosphate-buffered saline (PBS) for 30 min at room temperature (RT) and incubated with primary antisera (Table 1) in PBS-NDS for 18 h, at RT, followed by incubation with secondary antisera (Table 1) in PBS-NDS for 4 h, at RT. The sections were then incubated in an ABC reagent for 2 h, at RT, further soaked in 5 mg/ml diaminobenzidine (DAB)–HCl solution [pH 7.4, 5% (v/v) ammonium sulfate], and submerged in 0.3% (v/v) hydrogen peroxide solution in distilled water for 15–30 min. All reactions were monitored under a microscope. The DAB reaction was terminated by rinsing with 0.01 M PBS. Finally, the sections were mounted, air dried, dehydrated in a graded series of diluted ethanol, cleared in xylene, and coverslipped.

**Table 1:** Antisera used for immunofluorescence staining and DAB reactions.

	Group	Primary antisera	Secondary antisera	Tertiary antisera
Double staining	GFAP/NeuN	Mouse anti-GFAP	Alexa 488-donkey anti-mouse	
		(1:500, MAB3402, Millipore, USA)	(1:500, 12943, Cellsignal, USA)	
		Rabbit anti-NeuN	Alexa594-donkey rabbit	
		(1:500, 12943, Cellsignal, USA)	(1:500, A21207, Invitrogen, USA)	
	PHA-L/PV	Rabbit anti-PHA-L	Alexa488-donkey anti-rabbit	
		(1:500, CA9440, EY Laboratories, USA)	(1:500, A21206, Invitrogen, USA)	
		Mouse anti-PV	Alexa594-donkey anti-mouse	
		(1:500, P3088, Sigma, USA)	(1:500, A21203, Invitrogen, USA)	
	PHA-L/FG	Rabbit anti-PHA-L	Alexa488-donkey anti-rabbit	
		(1:500, CA9440, EY Laboratories, USA)	(1:500, A21206, Invitrogen, USA)	
		Guinea pig anti-FG	Alexa594-donkey anti-Guinea pig	
		(1:500, NM-101, Protos Biotech, USA)	(1:500, 706-545-148, Jackson immuno, USA)	
	PHA-L/VGluT2	Rabbit anti-PHA-L	Alexa594-donkey anti-rabbit	
	uT2	(1:500, CA9440, EY Laboratories, USA)	(1:500, A21207, Invitrogen, USA)	
		Guinea pig anti-VGLUT2	Alexa488-donkey anti-Guinea pig	
		(1:500, 135404, Synaptic Systems, Germany)	(1:500, 706-545-148, Jackson immuno, USA)	
Triple staining	PHA-L/FG/FOS	Rabbit anti-PHA-L	Alexa 488-donkey anti-rabbit	
		(1:500, CA9440, EY Laboratories, USA)	(1:500, A21206, Invitrogen, USA)	
		Guinea pig anti-FG	Alexa 594-donkey anti-Guinea pig	
		(1:500, NM-101, Protos Biotech, USA)	(1:500, 706-545-148, Jackson Immuno, USA)	
		Mouse anti-Fos	Alexa 647-donkey anti-mouse	
		(1:500, ab11959, Abcam, USA)	(1:500, A31571, Invitrogen, USA)	
DAB reactions	FG	Rabbit anti-FG	Biotin-donkey anti-rabbit	ABC kit
		(1:500, A153-I, Millipore, USA)	(1:500, AP182B, Millipore, USA)	(1:200, PK-6101, Vector Labs, USA)
	PHA-L	Rabbit anti-PHA-L	Biotin-donkey anti-rabbit	
		(1:500, CA9440, EY Laboratories, USA)	(1:500, AP182B, Millipore, USA)	ABC kit
				(1:200, PK-6101, Vector Labs, USA)

*DAB, diaminobenzidine; FG, Fluoro-Gold*.

Double or triple immunohistochemical staining for FG/Fos/PHA-L, PHA-L/parvalbumin (PV), PHA-L/FG, PHA-L/VGluT2, and GFAP/NeuN was performed. The primary and secondary antisera are listed in Table 1. The sections were incubated with primary antisera in the antibody dilution medium (pH 7.4) consisting of 0.01 M PBS (pH 7.4) with 5% (v/v) normal donkey serum (PBS-NDS), 0.3% (v/v) Triton X-100, 0.05% (w/v) sodium azide (NaN_3_), and 0.25% (w/v) carrageenan for 24 h, at RT. The sections were then incubated with the secondary antisera in the antibody dilution medium for 5 h, at RT. The sections were then mounted and coverslipped. Finally, the sections were imaged with a confocal laser scanning microscope (CLSM, FV1000, Olympus, Tokyo, Japan).

### FISH With FG Immunohistochemical Staining

Double distilled water, 0.1 M PB, and 0.01 M PBS were preprocessed with 0.1% (v/v) diethyl pyrocarbonate (DEPC, V900882, Sigma, St. Louis, MO, USA) for 8 h and autoclaved. Three rats were injected with 4% FG solution in the BLA. After 7 d, the rats were perfused transcardially with 150 ml PBS (0.01 M, pH 7.4), followed by 500 ml 4% paraformaldehyde in sodium PB (0.1 M, pH 7.4). The brains were removed and postfixed for 72 h, all in the same fixative, and then immersed in 30% sucrose in 0.1 M PB for 48 h at 4°C. The brains were sectioned into 40-μm slices with a cryostat. *In situ* hybridization of digoxigenin-labeled VGluT2 mRNA probe and tyramide signal amplification were performed as previously described (Nakamura et al., 2007). On the first day, the sections were incubated in 2% (v/v) hydrogen peroxide in PB for 10 min to quench endogenous peroxidase activity. Pre-hybridization was performed for 1 h at 58°C in hybridization solution [50% (v/v) formamide, 25% (v/v) 20× standard saline citrate (SSC), 2% (w/v) blocking reagent (Roche Diagnostics), 0.1% (w/v) *N*-lauroylsarcosine (NLS), and 0.1% (w/v) sodium dodecyl sulfate (SDS)]. Then, digoxigenin-labeled VGluT2 riboprobe (final concentration 1 ng/μl) was added for 20 h at 58°C. On the second day, the sections were incubated with a mixture of 1:500 diluted rabbit anti-FG antibody (1:500, NM-101, Protos Biotech, New York, NY, USA) and 1:1,500 diluted peroxidase-conjugated anti-digoxigenin sheep antibody (11-207-733-910, Roche Diagnostics, Basel, Switzerland) in TBS solution [0.1 M Tris–HCl (pH 7.5), 0.9% (w/v) NaCl, 1% (w/v) blocking reagent] for 18 h, at RT. On the third day, biotinylated tyramine amplification was performed in a mixture containing 1.25 μM biotinylated tyramine, 1 mg/ml glucose oxidase, 2 mg/ml beta-D-glucose, and 1% (w/v) bovine serum albumin in 0.1 M PB for 30 min at RT. The sections were incubated with 1:500 diluted FITC-Avidin (A-2001; Vector, Burlingame, CA, USA) and 1:500 Alexa 594-donkey rabbit antibody (1:500, A21207, Invitrogen, Carlsbad, CA, USA) in TNT solution [0.05% (v/v) Tween-20, 0.1 M Tris–HCl (pH 7.5), 0.15 M NaCl] for 3 h. There were no hybridization signals in the sections using the sense probe.

### Statistical Analysis

Data are reported as the mean ± standard deviation. The analysis of variance of the repeated measurement design based on the statistical software IBM SPSS 26.0. *P* < 0.05 indicated that the difference was statistically significant. According to the Mauchly spherical test results (*P* < 0.001), spherical symmetry was not met. Consequently, the subsequent analysis used the correction coefficient by the Greenhouse–Geisser method. The individual effects were then compared at different time points because of the interaction between the pain threshold detection at different time points for the different groups. The immunofluorescence images were photographed with a confocal laser scanning microscope (FV1000, Olympus, Tokyo, Japan). Six images (100 × 100 μm) were randomly chosen to count the number of varicosities in each subregion of the BLA, as described previously (Tasset et al., 2012). The counting of the cells or of the varicosities was performed using ImageJ image analysis software (National Institutes of Health, Bethesda, MD, USA).

## Results

### KA Lesion Induced Neuronal Loss and Astrocytosis in the CM

**Figure 1:**
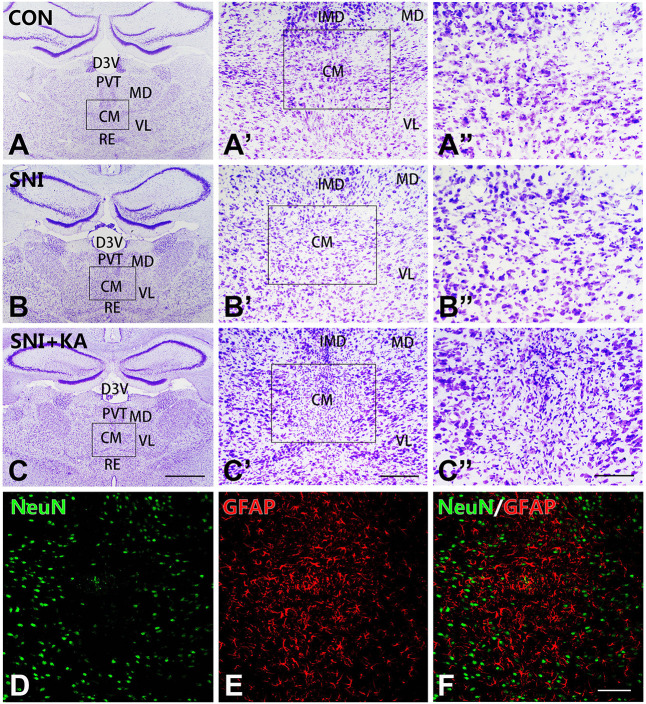
The morphology and reactive astrocytosis of the CM in different groups. Representative Nissl staining images showing Nissl bodies in the CM regions in the CON **(A,A’,A”)**, spared nerve injury (SNI) **(B,B’,B”)**, and SNI + KA **(C,C’,C”)** group rats. The framed areas in panels **(A–C)** are magnified in panels **(A’–C’)** and then again in **(A”–C”)**, respectively. Bars = 1 mm in panels **(A–C)**, 200 μm in panels **(A’–C’)**, and 100 μm in panels **(A”–C”)**. **(D–F)** Double immunohistochemical staining of NeuN (**D**; green) and GFAP (**E**; red) in the SNI + KA group of rats only. **(F)** Merged image of panels **(D,E)**. Bar = 200 μm. CM, central medial thalamic nucleus; D3V, dorsal third ventricle; IMD, intermediodorsal thalamic nucleus; MD, mediodorsal thalamic nucleus; PVT, paraventricular thalamic nucleus; VL, ventrolateral thalamic nucleus.

Nissl staining was used to assess the cyto-architecture changes in the CM in different groups. After the microinjection, the number of neurons in the CM in the SNI + KA group (Figures 1C,C’,”) significantly decreased, and the remaining neurons also lost their normal morphology compared to those in the CON (Figures 1A,A’,A”) and SNI group rats (Figures 1B,B’,B”). The KA lesion induced reactive astrocytosis and a decrease in the number of neurons in the CM, as revealed by increased GFAP and decreased NeuN expression (Figures 1D–F). The results showed significant neuronal loss and astrocytosis after KA injection into the CM, which indicated successful lesion of CM.

### CM Lesion Alleviated the Mechanical Allodynia in SNI Rats

**Figure 2:**
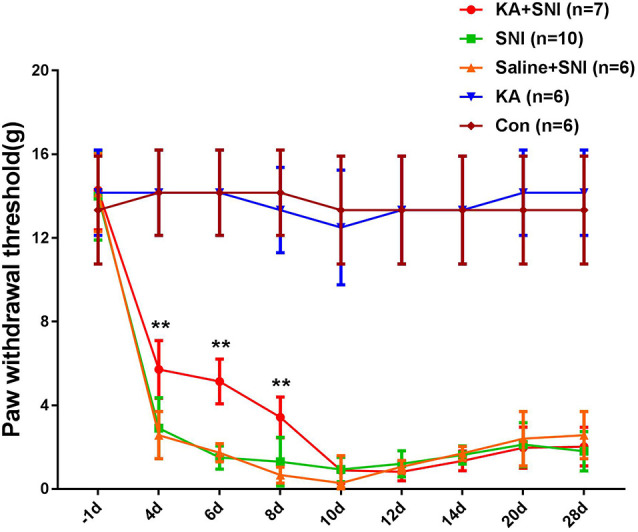
Time course of the neuropathic pain behavioral changes in five groups of rats. The rats of the SNI + KA group showed significant attenuation of allodynia and hyperalgesia at 4, 6, and 8 days compared to that in the SNI and SNI + SALINE groups. **P < 0.01.

To investigate the role of the CM in the maintenance of neuropathic pain, we performed SNI surgery following KA injection into the CM. The mechanical PWT was measured with von Frey filaments (Figure 2). The rats in SNI and SNI + Saline groups showed typical mechanical hyperalgesia, as described previously (Pertin et al., 2012). However, compared with the SNI and SNI + SALINE groups, the SNI + KA group showed elevated PWT at 4, 6, and 8 days after SNI (*P* < 0.01). Typical mechanical hyperalgesia returned after day 10. Interestingly, the PWT in the KA group did not differ from that of the CON group, suggesting that the KA group showed no mechanical hyperalgesia. These data revealed that CM lesion had no effect on nociception in normal rats. Under SNI conditions, the mechanical hyperalgesia was alleviated after the KA lesion of CM, indicating that CM might play an important role on the formation of neuropathic pain.

### The CM Received Projections From the vlPAG and Sent Projections to the BLA

**Figure 3:**
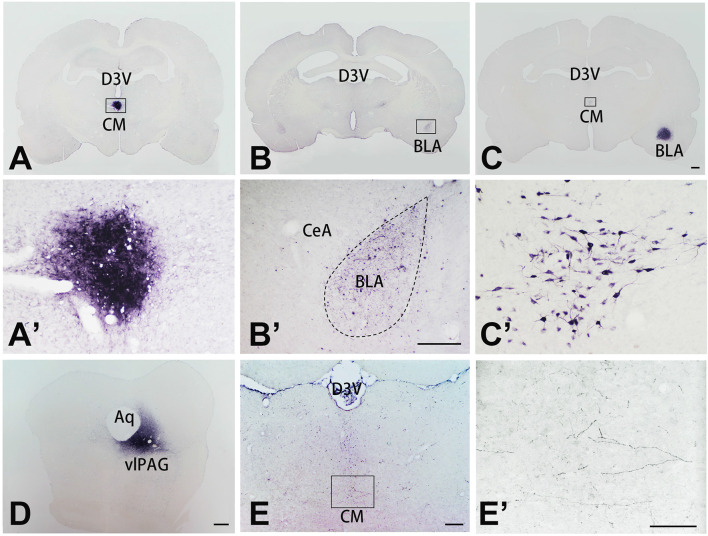
The tracer injection site in representative coronal sections of the CM, BLA, vlPAG, and relevant tracing sections. The tracer was visualized with the diaminobenzidine (DAB) reaction and enhanced with ammonium sulfate. **(A)** Coronal section showing the injection site of PHA-L in the CM; the framed area is magnified in panel **(A’)**. **(B)** Coronal section showing labeled fibers and terminals in the BLA, projected from the CM; the framed area is magnified in panel **(B’)**. **(C)** Coronal section showing the locations of FG injections in the BLA. **(C’)** Retrogradely labeled neurons are seen in the CM, magnified from the framed area in **(C)**. Bar = 1 mm in panels **(A–C)**. **(D)** Coronal section showing the injection site of PHA-L in the vlPAG. Bars = 1 mm in panel **(D)**, 100 μm in panels **(A’,B’)**. **(E)** Coronal section showing anterogradely labeled fibers and terminals in the CM, projecting from the vlPAG nucleus; the framed area is magnified in panel **(E’)**. Bars = 200 μm in panel **(E)**. 100 μm in panels **(C’,E’)**. Aq, aqueduct; BLA, basolateral amygdaloid nucleus anterior part; CeA, central amygdaloid nucleus; CM, central medial thalamic nucleus; D3V, dorsal third ventricle; vlPAG, ventrolateral periaqueductal gray matter.

To further investigate the connective relationship between CM and vlPAG as well as amygdala, we performed tract-tracing experiments. Anterograde tracer PHA-L was iontophoretically injected into the CM (Figures 3A,A’) and resulted in dense labeling in the bilateral BLA (Figures 3B,B’). To verify this CM-BLA projection, the retrograde tracer FG was then injected into the unilateral BLA (Figure 3C). FG injection into the right BLA produced dense retrograde labeling of a band of neurons located in the CM (Figures 3C,C’ and Supplementary Figure S1). Subsequently, the anterograde tracer PHA-L was iontophoretically injected into the vlPAG (Figure 3D) to detect the vlPAG–CM projection. The results showed that PHA-L-labeled fibers and terminals were observed in the CM (Figures 3E,E’). These tract-tracing data revealed that the CM received afferent fibers from the vlPAG and sent projections to the BLA.

### The CM-BLA Projecting Pathway Was Primarily an Excitatory Projection

**Figure 4:**
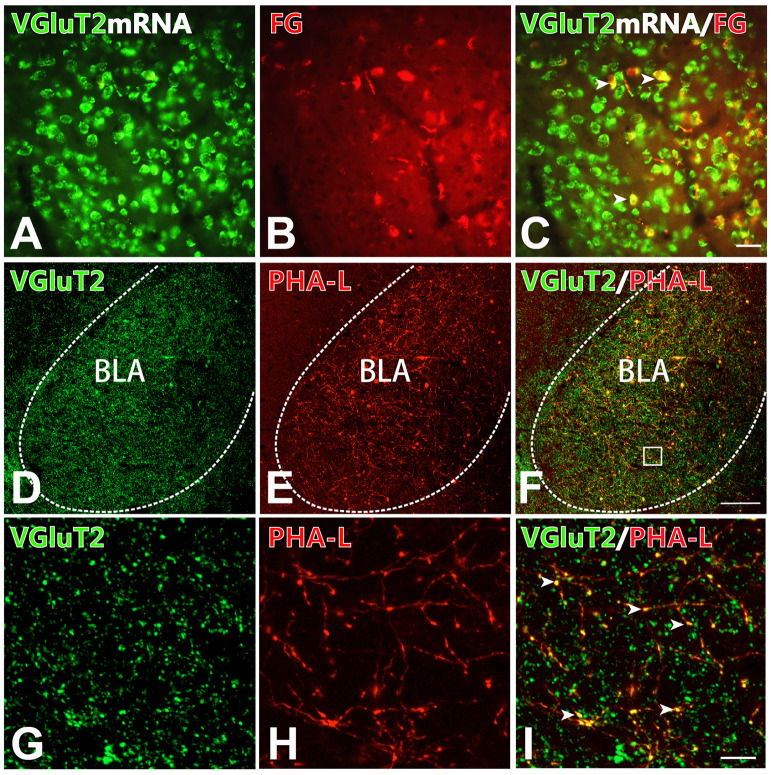
BLA-projection neurons in the CM nucleus are mainly VGluT2-ir glutamatergic neurons. **(A–C)** After Fluoro-Gold (FG) injection into the BLA, cell bodies co-expressing VGluT2 mRNA signals (**A**; green) and FG-ir (**B**; red) in the CM. **(C)** Merged image of **(A,B)**. Arrows indicating the FG-ir cell bodies co-expressing VGluT2 mRNA signals. Bar = 100 μm in panels **(A–C)**. **(D–I)** After PHA-L injection into the CM, fluorescence photomicrographs showing axon terminals co-expressing VGluT2 (**D**; green) and PHA-L (**E**; red) in the BLA. **(F)** Merged image of panels **(D,E)**. Bar = 100 μm in panels **(D–F)**. The framed area in panel **(F)** is magnified in panels **(G–I)**. Arrowheads in panel **(I)** indicate PHA-L-ir axon terminals co-expressing VGluT2-ir varicosities. Bar = 30 μm in panels **(G–I)**. BLA, basolateral amygdaloid nucleus, anterior part.

To detect the neurochemical properties of BLA-projecting neurons within the CM FISH histochemistry combined with retrograde tract-tracing was performed. After FG injection into the BLA retrogradely labeled FG-immunoreactive neurons were distributed throughout the CM and 96.90 ± 0.61% (1,749/1,805) of FG-labeled CM neurons were double-labeled with VGluT2 mRNA hybridization signals (Figures 4A–C; Table 2). On the side contralateral to the FG injection, few FG-labeled cells were scattered in the CM. Moreover, after iontophoretical injection of anterograde tracer PHA-L into the CM, immunofluorescent histochemical staining was conducted to detect the fibers and terminals in the BLA. We observed that PHA-L-labeled varicose and punctate terminals arising from projection neurons in the CM were double-labeled with VGluT2-immunoreactive (ir) varicosities (Figures 4D–I). Approximately 98.04 ± 1.10% (4,964/5,066) of the PHA-L-labeled fibers showed VGluT2-ir expression (Table 3). These results indicated that the neurochemical properties of BLA-projecting neurons in the CM were mainly glutamatergic VGluT2-ir neurons.

**Table 2:** Numbers and percentages of FG/VGluT2 mRNA double-labeled neurons in the CM*.

	FG	FG + VGluT2 mRNA	FG + VGluT2 mRNA/FG (%)
Rat 1	651	629	96.62
Rat 2	604	583	96.52
Rat 3	550	537	97.64
Rat 4	635	621	97.80
Rat 5	601	585	97.34
Rat 6	673	657	97.62
Rat 7	578	559	96.71
Rat 8	590	563	95.42
Total	4882 ± 40	4734 ± 41	96.96 ± 0.80

*The number was counted in six sections from a series of 40-μm-thick sections. FG, Fluoro-Gold.

**Table 3:** Numbers and percentages of FG/VGluT2 double-labeled varicosities in the BLA*.

	PHA-L	PHA-L + VGluT2	PHA-L + VGluT2/PHA-L (%)
Rat 17	1,768	1,720	97.29
Rat 18	1,553	1,542	99.29
Rat 19	1,745	1,702	97.54
Rat 20	1,663	1,653	99.40
Rat 21	1,597	1,584	99.19
Rat 22	1,607	1,592	99.07
Rat 23	1,723	1,696	98.43
Rat 24	1,772	1,748	98.65
Total	13,428 ± 85	13,237 ± 74	98.61 ± 0.80

*The number was counted in six sections from a series of 40-μm-thick sections.

### The CM-BLA Projecting Fibers and Terminals Formed Close Connections With Both Excitatory and Inhibitory Neurons in the BLA

**Figure 5:**
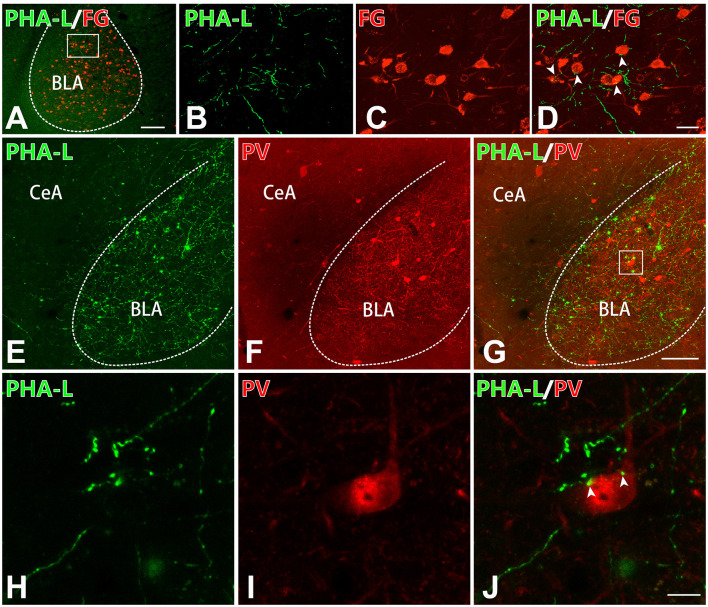
The CM–BLA projecting fibers formed close connections with both excitatory and inhibitory neurons in the BLA. **(A)** Low-magnification fluorescence photomicrographs showing PHA-L-ir axon terminals (green) forming close connections with an FG-ir neuronal cell body (red) in the BLA. Bar = 1 mm in panel **(A)**. The framed area in panel **(A)** is magnified in panels **(B–D)**. **(D)** Merged image of panels **(B,C)**. Arrowheads indicate the close connection sites. Bar = 30 μm in panels **(B–D)**. **(E–G)** PHA-L-ir axon terminals (**E**, green) forming close connections with a PV-ir neuronal body (**F**, red). **(G)** Merged image of panels **(E,F)**. Bar = 100 μm in panels **(E–G)**. **(H–J)** High-magnification images of the framed area in panel **(G)** showing close connections between PHA-ir axon terminals (**H**, green) and a PV-ir neuronal body (**I**, red) in the BLA. **(J)** Merged image of panels **(H,I)**. Arrowheads indicate the close connection sites. Bar = 10 μm in panels **(H–J)**. BLA, basolateral amygdaloid nucleus, anterior part; CeA, central amygdaloid nucleus.

To detect whether the projecting fibers and terminals from the CM formed close connections with the excitatory or inhibitory neurons in the BLA, immunofluorescence histochemistry combined with anterograde tract-tracing methods were performed in the present experiment. On the basis of previous work that proved that the excitatory glutamatergic neurons in the BLA primarily send projection to the mPFC (Imperatore et al., 2015), FG was injected into the mPFC. Thus, FG retrogradely labeled neurons in the BLA would represent excitatory neurons. It has been reported that the inhibitory neurons in the BLA were mainly PV-ir GABAergic neurons (Prager et al., 2016). After injection of PHA-L into the CM and FG into the mPFC, we observed that the PHA-L-ir fibers and terminals formed close connections with FG-ir glutamatergic neurons in BLA (Figures 5A–D). Moreover, after PHA-L injection into the CM, PHA-L-ir terminals (Figures 5E,H) were also found to form close connections (Figures 5G,J) with PV-ir inhibitory interneurons (Figures 5F,I) in the BLA. These data showed that VGluT2-ir glutamatergic projection neurons in the CM could project and terminate onto both glutamatergic excitatory neurons and PV-ir GABAergic inhibitory neurons in the BLA.

### The vlPAG-CM-BLA Pathway Was Involved in the Modulation of Neuropathic Pain in Rats

**Figure 6:**
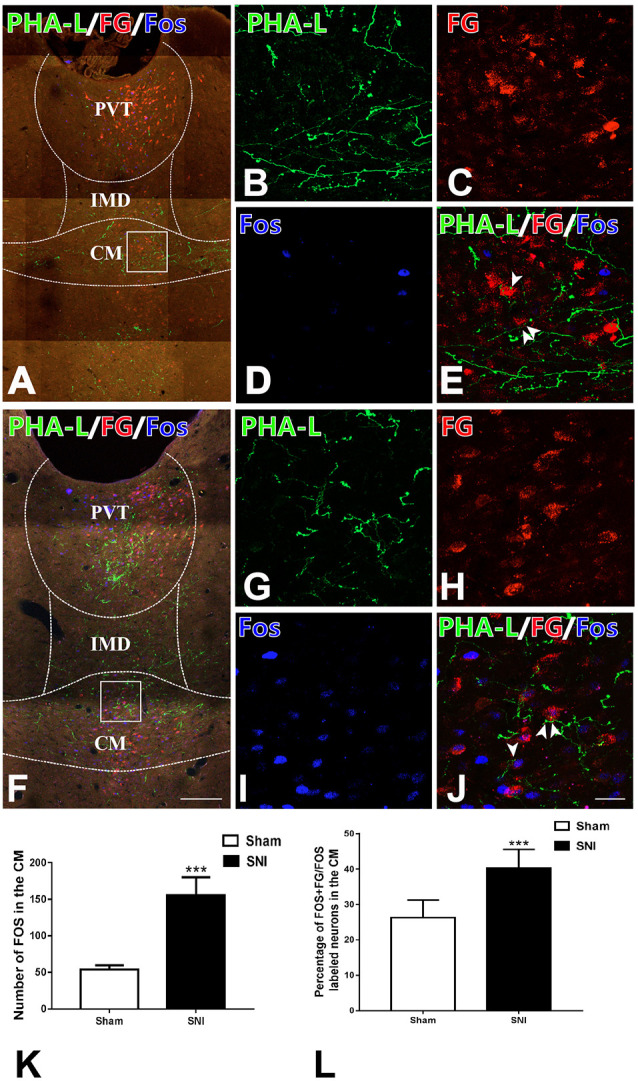
Fos staining and the connections between anterogradely labeled PHA-L-ir axon terminals from the vlPAG and retrogradely labeled FG-ir neurons from BLA in the CM of the sham and the SNI groups. **(A)** Low-magnification fluorescence photomicrographs showing that PHA-L-ir neural terminals (green) formed close connections with neuronal cell bodies labeled with FG (red) and expressing Fos-ir nucleus (blue) in the sham-operated group. The square-framed area in panel **(A)** is magnified in panels **(B–E)**. **(E)** Merged image from panels **(B–D)**. Arrowheads indicate the close connection sites. **(F)** Low-magnification fluorescence photomicrographs showing that PHA-L-ir neural terminals (green) formed close connections with neuronal cell bodies labeled with FG and Fos (blue) in the SNI group. The square-framed area in panel **(F)** is magnified in panels **(G–J)**. **(J)** Merged image from panels **(G–I)**. Arrowheads indicate the close connection sites. **(K)** The number of Fos-ir neurons in the CM; ***P < 0.001. **(L)** The percentages of Fos/FG double-labeled neurons among FG-ir neurons in the CM; ***P < 0.001. Bars = 200 μm in panels **(A,F)**; 30 μm in panels **(B–E,G–J)**. CM, central medial thalamic nucleus; IMD, intermediodorsal thalamic nucleus; PVT, paraventricular thalamic nucleus.

In order to prove that the vlPAG–CM–BLA pathway was involved in the modulation of neuropathic pain, we used anterograde and retrograde tract-tracing methods combined with Fos staining to find out whether the CM relaying neurons in the vlPAG-CM-BLA pathway were activated under SNI conditions. After injecting FG into BLA and PHA-L into vlPAG in sham-operated and SNI rats, triple-immunofluorescence histochemical staining was conducted. The distribution profiles of FG-ir, PHA-L-ir, and Fos-ir within the CM were observed and calculated in the sham-operated (Figures 6A–E) and SNI (Figures 6F–J) groups. The anterogradely labeled PHA-L-ir fiber varicose and punctate terminals made close contact with retrogradely labeled FG-ir neurons within the CM (Figures 6E,J), suggesting the existence of the vlPAG-CM-BLA pathway. Moreover, the expression of Fos in the CM neurons was obviously increased in the SNI group compared with those of the sham group (Figures 6D,I). In addition, FG-labeled BLA-projecting neurons that contacted with PHA-L-labeled projecting fibers from the vlPAG showed elevated Fos expression in the CM in the SNI group rats compared with those of sham rats (Figures 6K,L; Table 4). As shown in Table 4, the numbers of Fos-labeled neurons in the CM were 160 ± 6 and 418 ± 21 in the sham and SNI group rats, respectively (*P* < 0.01). The numbers of Fos/FG double-labeled neurons in the CM were 39 ± 2 and 159 ± 7 in the sham and SNI group rats, respectively. Furthermore, 2.48 ± 0.44% and 10.10 ± 1.29% of FG-ir neurons in the CM were Fos/FG double-labeled in the sham and SNI group rats, respectively, while 24.38 ± 1.37% and 38.04 ± 1.53% of Fos-ir neurons in the CM were Fos/FG double-labeled in the sham and SNI group rats, respectively (*P* < 0.001). These results indicated that the vlPAG–CM–BLA pathway might be involved in the transmission and modulation under the neuropathic pain conditions.

**Table 4:** Numbers and percentages of Fos/FG double-labeled neurons in the CM*.

		Fos	FG	Fos + FG	Fos + FG/FG (%)	Fos + FG/Fos (%)
Sham	Rat 1	46	550	11	2.00	23.91
	Rat 2	58	531	15	2.82	25.86
	Rat 3	56	489	13	2.66	23.21
	Rat 4	54	486	20	4.12	37.04
	Rat 5	47	501	12	2.40	25.53
	Rat 6	60	452	17	3.76	28.33
	Rat 7	49	527	10	1.90	20.41
	Rat 8	61	477	16	3.35	26.23
	Total	431 ± 5.89	4,013 ± 32	114 ± 3.37	2.88 ± 0.81	26.32 ± 4.93
SNI-Fos	Rat 9	128	495	51	10.30	39.84
	Rat 10	163	542	61	11.25	37.42
	Rat 11	127	540	47	8.70	37.01
	Rat 12	174	478	71	14.85	40.80
	Rat 13	162	487	52	10.68	32.10
	Rat 14	154	501	71	14.17	46.10
	Rat 15	136	463	55	11.88	40.44
	Rat 16	199	497	97	19.52	48.74
	Total	1243 ± 25^#^	4,003 ± 28	505 ± 16	12.67 ± 3.42	40.31 ± 5.24^#^

*The number was counted using four sections from a series of 40-μm-thick sections. FG, Fluoro-Gold. ^#^P < 0.001.

**Figure 7:**
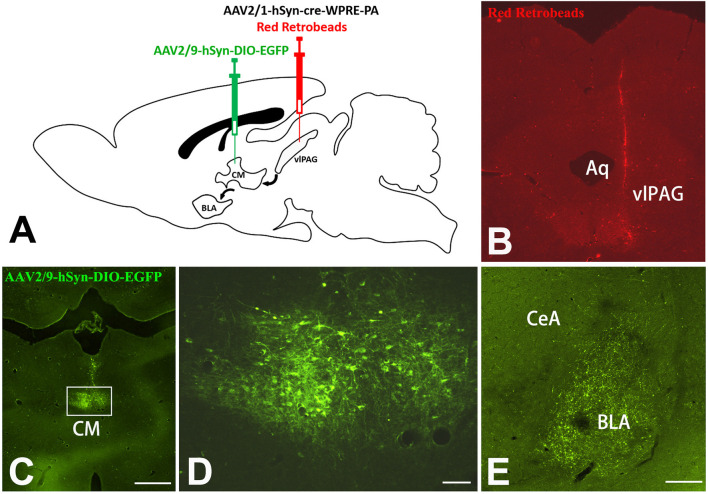
Cre-dependent anterograde trans-monosynaptic virus tracing of vlPAG-CM-BLA pathway. **(A)** Scheme of the two-step virus tracing. **(B)** AAV2/1-hSyn-Cre-WPRE-PA and Red Retrobeads were mixed and injected into the right vlPAG, and the injection range was concentrated in the vlPAG. **(C)** AAV2/9-hSyn-DIO-eGFP was injected into the CM. Bar = 500 μm in panels **(B,C)**. The framed area in panel **(C)** is magnified in panel **(D)**. Bar = 50 μm in panel **(D)**. **(E)** Image shows eGFP-labeled fibers and terminals in the BLA. Bar = 200 μm in **(E)**. CM, central medial thalamic nucleus; BLA, basolateral amygdaloid nucleus, anterior part; vlPAG, ventrolateral periaqueductal gray matter; CeA, central amygdaloid nucleus; Aq, aqueduct.

A Cre-dependent anterograde trans-monosynaptic virus tracing method was used to further verify the vlPAG–CM–BLA pathway. The two-step tracing was used to examine the vlPAG–CM–BLA pathway (Figure 7A). The AAV2/1-hSyn-Cre-WPRE-PA virus was injected into the rats’ vlPAG so that the Cre enzyme could spread anterogradely and monosynaptically into the soma of the neurons in the CM. Red Retrobeads were mixed with AAV2/1-hSyn-Cre-WPRE-PA at a ratio of 1:1 to label the injection site (Figure 7B). Red Retrobeads with a final concentration of 1:1,000 had almost no traceability. The red color was used to help identify whether the colorless AAV2/1-hSyn-Cre-WPRE-PA virus was injected accurately into vlPAG. The placement of the end of the red needle way was observed exactly inside the vlPAG. Meanwhile, Cre-dependent anterograde AAV2/9-hSyn-DIO-eGFP was injected into the CM to identify the downstream targets of the vlPAG–CM pathway (Figures 7C,D). The results revealed that abundant fibers and terminals could be observed in the BLA (Figure 7E), which implied that BLA-projecting CM neurons received projections from the vlPAG.

## Discussion

This study, to the best of our knowledge, is the first to demonstrate the existence of the vlPAG–CM–BLA pathway, and such a pathway might be involved in neuropathic pain in rats. The present findings extend previous reports about the sources of BLA pain information and provide a valuable starting point for identifying the specific role of CM under neuropathic pain conditions.

### Lesion of the CM Alleviated Mechanical Allodynia in SNI Rat

As a traditional, functional, “nonspecific” nucleus for maintaining arousal, the CM has many “pain-specific” functions that play a major role in regulating arthritis pain and visceral pain. These characteristics have been shown using electrophysiology and immunohistochemistry methods (Dostrovsky and Guilbaud, 1990; Lazovic et al., 2005). However, no behavioral evidence demonstrates the participation of CM in neuropathic pain. In the present study, a KA-induced excitotoxic lesion of the CM alleviated mechanical hyperalgesia in SNI rats. The increased Fos expression under the neuropathic pain in the CM indicated that CM contains pain-specific neurons. This finding is in agreement with that of previous reports in which the noxious inputs elevated p-ERK and Fos expression within the CM in rats (Castro-Alamancos, 1997; Ter Horst et al., 2001; Zhang et al., 2009). Moreover, many input and output brain regions of CM are involved in the regulation of pain. Prior research has revealed that the CM receives serotoninergic projection fibers from the PAG and the dorsal raphe nucleus, which, when electrically stimulated, could induce potent analgesia (Sim and Joseph, 1992). Meanwhile, the projection regions of the CM include the ACC, mPFC (Vertes et al., 2012), and BLA, which have also been demonstrated to be important in pain regulation (Li et al., 2010; Zhang et al., 2015). These results indicate that the CM might play an important role as a pain relay station. In addition, in the present study, the majority of neurons of the CM were glutamatergic VGluT2-ir neurons, a finding that was exhibited in a previous report (Barroso-Chinea et al., 2007). A lesion of the VGluT2-ir neurons in the CM can reduce the excitatory output transmission of nociceptive information and alleviate the mechanical PWT in SNI rats. Similarly, a significant analgesia of hot pain was obtained by microinjection of ketoprofen into the CM, and the spontaneous activity of thalamic neurons can be reduced (Braga, 1990; de Beaurepaire et al., 1990). To date, our results are the first observations of behavioral changes induced by a CM lesion in a rat neuropathic pain model.

Allodynia and hyperalgesia can last for many weeks in SNI model rats (Pertin et al., 2012). In the present study, the analgesic effect of a CM lesion revealed by PWT elevation in rats was observed in days 4–8 after SNI, suggesting that CM was involved in the maintenance of neuropathic pain. However, this effect disappeared 10 days after SNI surgery. Several similar nucleus lesion studies (recurrence of neuropathic pain behaviors) also reported similar phenomena (Al Amin et al., 2004; Saadé et al., 2006). Astrocyte activation and reactive gliosis were observed after a KA-induced excitotoxic lesion in the present work, which might limit the lesion range to promote CNS regeneration and reduce pathological manifestations of pain (Pekny et al., 2014). Conversely, such reversible attenuation of mechanical hyperalgesia may be due to plasticity changes and compensation of the pain matrix and finally lead to recovery of PWT (Saadé et al., 2007). Taken together, these data indicated that the CM plays an essential role in transmitting pain signals under neuropathic pain conditions.

### BLA Receives Pain Information From the CM–BLA Pathway

Previous evidence showed that the BLA is an important pain-processing region of the brain and receives nociceptive information mainly from cortical systems, including ACC, mPFC, and insula cortex (Shi and Davis, 1999; Kiritoshi and Neugebauer, 2018; Liu et al., 2019). However, few studies focused on the ILN. Our findings demonstrated that Fos/FG double-labeled neurons and the ratio of Fos + FG/Fos in the CM both increased under neuropathic pain conditions. These data indicated that the projection neurons in the CM can transmit nociceptive information to the BLA. However, not all Fos-ir neurons were BLA-projecting neurons in the CM, indicating that some nociceptive information was transmitted through other neural pathways, such as the CM-ACC pathway (Shyu and Vogt, 2009).

The present study showed that VGluT2-ir glutamatergic neurons of the CM can activate both glutamatergic neurons and PV-ir GABAergic interneurons within the BLA through the CM-BLA pathway. The present data are consistent with the results of CM axon terminals forming asymmetric synapses with glutamatergic neurons in the BLA (Amir et al., 2019). However, in this report, the synapses of GABAergic neurons were not detected. These results might imply a complicated local inhibitory circuit within the BLA, since glutamatergic and GABAergic neurons have been exhibited to execute opposing effects in pain sensation (Zikopoulos et al., 2017). Disruption of the balance between excitatory and inhibitory localized circuitry within the amygdala may ultimately induce pain (Kiritoshi and Neugebauer, 2018). Therefore, clarifying the role of the CM–BLA pathway in nociception, especially interactions between glutamatergic and GABAergic neurons that receive CM projections in BLA, requires further research. Taken together, these data indicate that CM might participate in the maintenance of persistent pain through the output of excitatory nociceptive information to the BLA.

The rostral part of the CM targets the BLA of the amygdala, whereas the caudal part of CM fibers distributes throughout the amygdala terminating in the lateral, central, anterior cortical, and basomedial nuclei of the amygdala (Vertes et al., 2012). However, this report (Vertes et al., 2012) did not describe the scope of the rostral and caudal parts of the CM in detail or the projection of the middle area of the CM. Iontophoretically depositing PHA-L into the CM at the posterior Bregma −2.6 mm revealed that neurons in the middle area of the CM mainly project to the BLA. In the central amygdaloid nucleus, only a small number of terminals are close to the BLA. Present data show that the projection of the middle part of the CM is similar to the projection of the rostral part.

### The vlPAG–CM–BLA Pathway Is an Ascending Pain Pathway

Previous studies showed that vlPAG was involved in descending inhibitory or facilitatory pain modulatory pathways (Drake et al., 2016; Kim et al., 2018; Huang et al., 2019). Moreover, it has been proposed that an ascending pain pathway involving the vlPAG might exist (Krout and Loewy, 2000). If its descending pathway is transected, PAG stimulation would also exhibit pain modulation function in animals (Morgan et al., 1989). In addition, electrophysiological evidence showed that PAG stimulation can suppress the responses of thalamic nociceptive neurons (Andersen, 1986). Local cerebral injections of ketoprofen, a non-steroidal anti-inflammatory drug, at the PAG and CM induced a significant analgesic effect in hot pain model rats (de Beaurepaire et al., 1990). Thus, an ascending pain pathway may exist that involves the PAG and higher brain regions. The present data showed that these SNI-activating Fos-ir neurons in the CM received afferent axons from the vlPAG and projected efferent fibers to the BLA, suggesting the existence of the vlPAG–CM–BLA pathway that participated in neuropathic pain. In consequence, BLA receives top-down pain regulation not only from the cortical systems but also from the ascending vlPAG–CM–BLA pathway.

The present data showed the existence of a vlPAG–BLA direct pathway (see Supplementary Figure S2), in spite of relatively sparse projection fibers, which is consistent with a previous report (Ottersen, 1981). Two independent groups of neurons were found within the CM (see Supplementary Figure S3), which indicated that the CM–BLA pathway and CM–mPFC pathway have different functions. Compared to the direct vlPAG–BLA pathway, the indirect vlPAG–CM–BLA pathway may not only serve as an auxiliary pathway but also participate in the advanced functional regulation of neuropathic pain.

Our study also has many limitations. In the future, optogenetic methods and electrophysiological techniques will be used to confirm the role of the vlPAG–CM–BLA pathway in neuropathic pain, and the relative mechanisms.

In summary, CM could alleviate mechanical allodynia under neuropathic pain conditions and participate in the maintenance of persistent pain through the CM–BLA pathway. The ascending vlPAG–CM–BLA pathway might be involved in transmission and modulation of neuropathic pain in rats and may be a new pain information source for BLA. Understanding the hierarchical organization of the three subcortical nuclei can enhance our understanding of the entire pain network, especial within the brain cortex.

## Data Availability Statement

All datasets generated for this study are included in the article Supplementary Material.

## Ethics Statement

The animal study was reviewed and approved by The Ethics Committee of the Air Force Medical University. The ethical guidelines of the Research and Education of Air Force Medical University (Xi’an, China) were strictly followed during the experimental process to minimize the number of rats used as well as their pain and suffering in all experiments.

## Author Contributions

Y-QL, HL, and D-SL designed and oversaw the project and revised the final version of the manuscript. YS, JW, S-HL, and JG processed the surgery and performed the immunohistochemical staining. J-NL and Y-BC performed the data analysis. YS prepared the figures and drafted the initial manuscript. All authors listed have made a direct contribution to the work.

## Conflict of Interest

The authors declare that the research was conducted in the absence of any commercial or financial relationships that could be construed as a potential conflict of interest.
