# Supplementary material for: Involvement of the Ventrolateral Periaqueductal Gray Matter-Central Medial Thalamic Nucleus-Basolateral Amygdala Pathway in Neuropathic Pain Regulation of Rats
Source: Front Neuroanat. 2020 Jul 21;14:32. doi: 10.3389/fnana.2020.00032 (PMC7394700; doi:10.3389/fnana.2020.00032)
Supplement: Supplementary file 1 [file Presentation_1.zip › Legends of Supplementary Figure.docx]

Supplementary **Figure 1**. The distribution of FG in CM and the corresponding hippocampus area. The distribution of FG-labeled neurons in the CM from rostral to caudal, after FG injection into BLA. Bars = 100 μm in **(A’-F’)**. **(A-F)** Hippocampus area corresponding to the same brain slice of **(A’-F’).** Bars = 500 μm in **(A-F)**.

Supplementary **Figure 2.** The tract-tracing of the vlPAG-BLA pathway. **(A)** Coronal section showing the injection site of PHA-L in the vlPAG. **(B)** Coronal section showing PHA-L-labeled fibers and terminals in the BLA and CeA, projected from vlPAG. Bar = 100 μm in **B**. Arrows in **(B)** indicate PHA-L-ir axon terminals. **(C)** Coronal section showing the injection site of FG in the BLA. **(D)** Retrogradely FG-labeled neurons are seen in the DR and vlPAG. Bar = 500 μm in A, C, D. **(D’)** Magnified from the framed area of vlPAG in **(D)**. Arrows in **(D’)** indicate FG-labeled neurons. Bars = 50 μm in D’. Aq, aqueduct; BLA, basolateral amygdaloid nucleus, anterior part; CeA, central amygdaloid nucleus; vlPAG, ventrolateral periaqueductal gray matter.

Supplementary **Figure 3.** The tracer injection site in representative coronal sections of the BLA, Prl, and relevant tracing sections of CM. **(A)** Coronal section showing the injection site of FG in the BLA. **(B)** Coronal section showing the injection site of TMR in the Prl. Bars = 200 μm in A, B. **(C)** Coronal section showing the FG-labeled neurons in the CM. **(D)** Coronal section showing the TMR-labeled neurons in the CM. **(E)** Merged image of C and D. Bars = 100 μm in C-E. White and red arrows indicate FG-labeled and TMR-labeled neurons in the CM, respectively. BLA, basolateral amygdaloid nucleus, anterior part; CeA, central amygdaloid nucleus; CM, central medial thalamic nucleus; Prl, prelimbic cortex.
