# Supplementary figures and images for: Involvement of the Ventrolateral Periaqueductal Gray Matter-Central Medial Thalamic Nucleus-Basolateral Amygdala Pathway in Neuropathic Pain Regulation of Rats
Source: Front Neuroanat. 2020 Jul 21;14:32. doi: 10.3389/fnana.2020.00032 (PMC7394700; doi:10.3389/fnana.2020.00032)

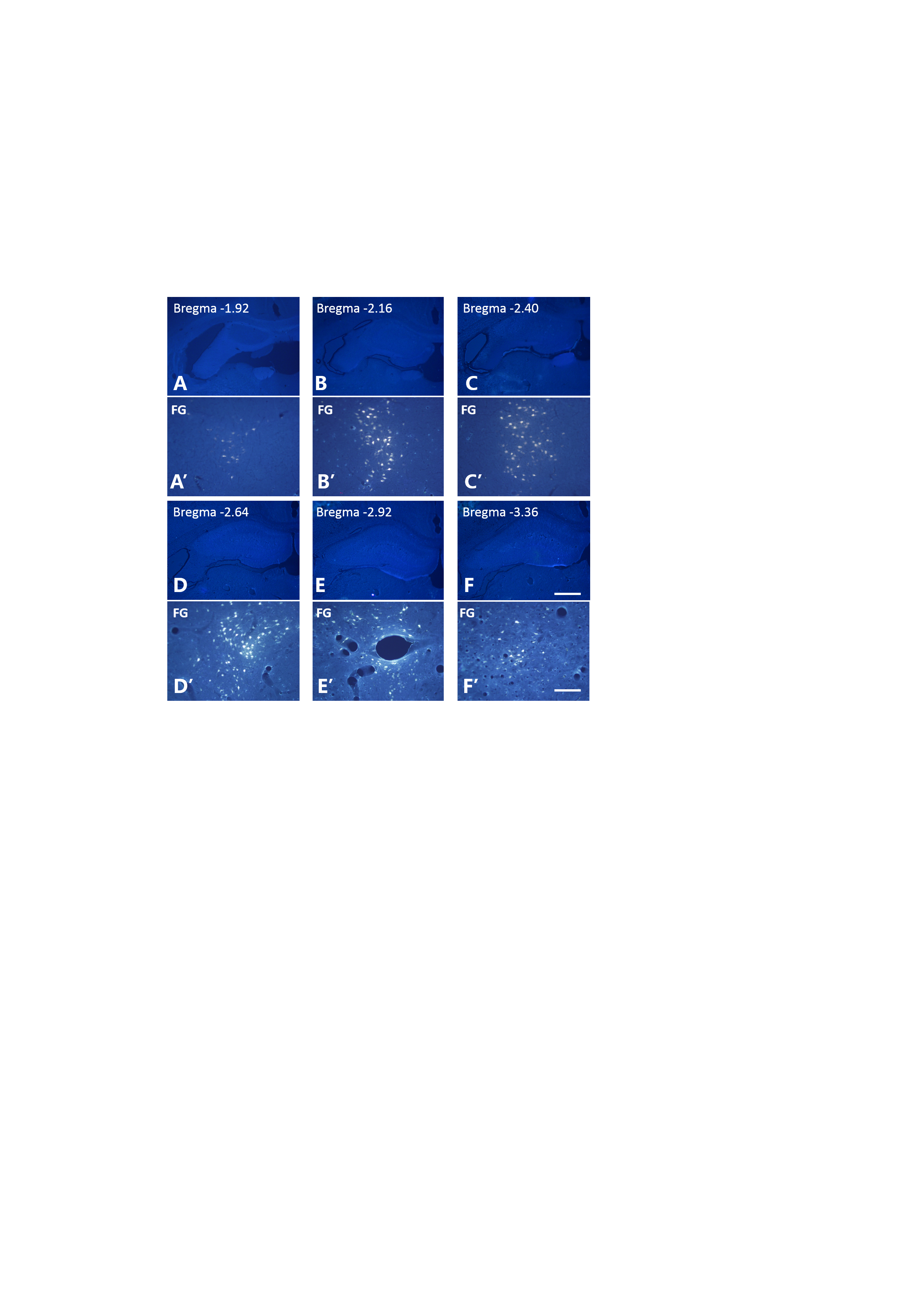

Supplement: Supplementary file 2 [file Image_1.jpg]

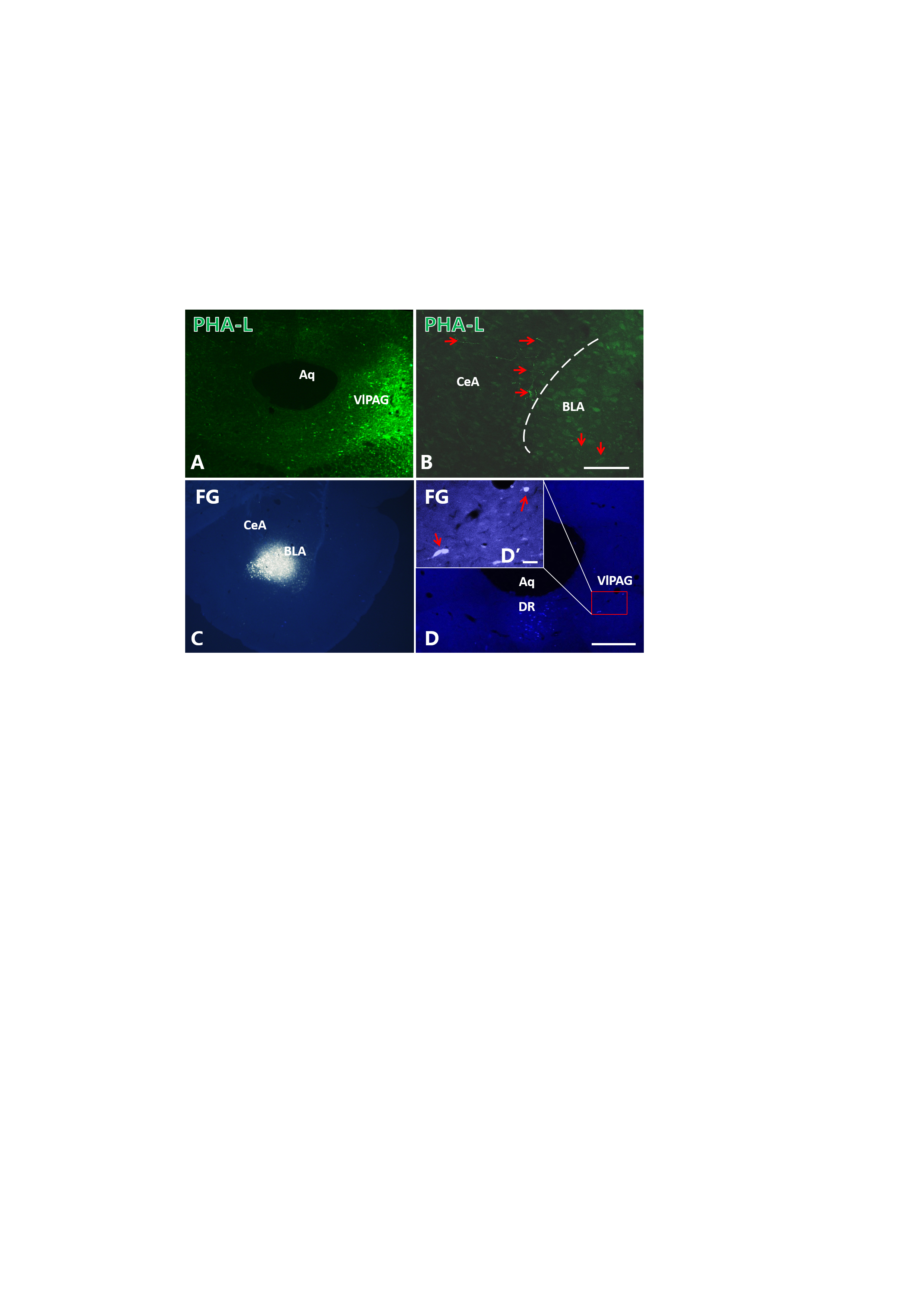

Supplement: Supplementary file 3 [file Image_2.jpg]

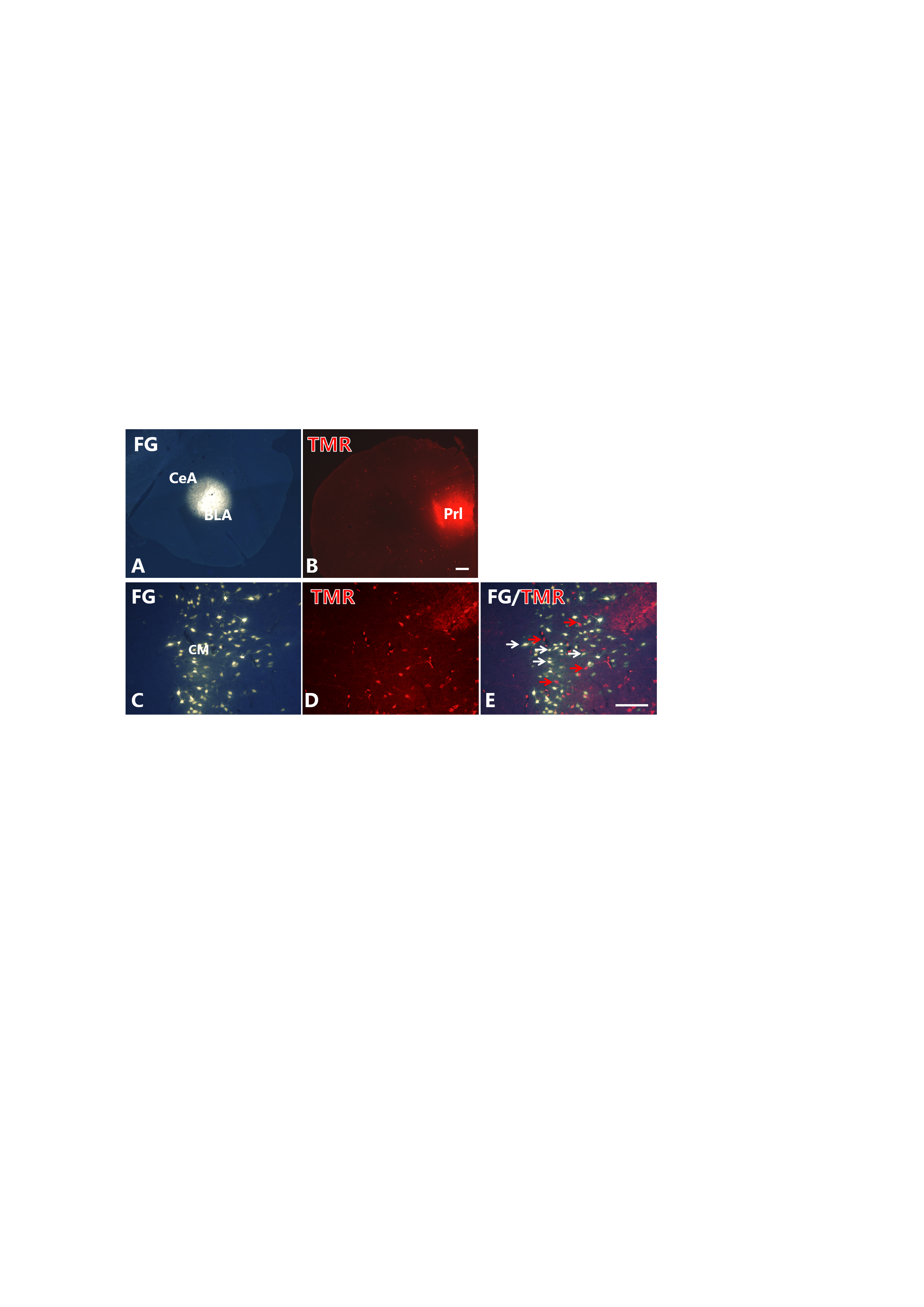

Supplement: Supplementary file 4 [file Image_3.jpg]
